# Primary stability of single-stage revision reconstruction of the anterior cruciate ligament in case of failure of dynamic intraligamentary stabilization depends on implant position during ACL repair

**DOI:** 10.1007/s00402-021-04088-4

**Published:** 2021-07-31

**Authors:** J. Glasbrenner, M. Fischer, M. J. Raschke, T. Briese, M. Müller, E. Herbst, C. Kittl, B. Schliemann, C. Kösters

**Affiliations:** 1 Department of Trauma, Hand and Reconstructive Surgery, University Hospital Münster, Albert-Schweitzer-Campus, Building W1, 48149 Münster, Germany; 2 Department of Traumatology and Orthopedics, Maria-Josef-Hospital Greven, Lindenstraße 29, 48268 Greven, Germany

**Keywords:** Dynamic intraligamentary stabilization, Anterior cruciate ligament repair, Anterior cruciate ligament revision surgery

## Abstract

**Introduction:**

The object of this study was to evaluate the primary stability of tibial interference screw (IFS) fixation in single-stage revision surgery of the anterior cruciate ligament (ACL) in the case of recurrent instability after ACL repair with dynamic intraligamentary stabilization (DIS), dependent on the implant position during DIS.

**Materials and methods:**

Tibial aperture fixation in ACL reconstruction (ACL-R) was performed in a porcine knee model using an IFS. Native ACL-R was performed in the control group (*n* = 15). In the intervention groups DIS and subsequent implant removal were performed prior to single-stage revision ACL-R. A distance of 20 mm in group R-DIS1 (*n* = 15) and 5 mm in group R-DIS2 (*n* = 15) was left between the joint line and the implant during DIS. Specimens were mounted in a material-testing machine and load-to-failure was applied in a worst-case-scenario.

**Results:**

Load to failure was 454 ± 111 N in the R-DIS1 group, 154 ± 71 N in the R-DIS2 group and 405 ± 105 N in the primary ACL-R group. Load-to-failure, stiffness and elongation of the group R-DIS2 were significantly inferior in comparison to R-DIS1 and ACL-R respectively (*p* < 0.001). No significant difference was found between load-to-failure, stiffness and elongation of R-DIS1 and the control group.

**Conclusion:**

Primary stability of tibial aperture fixation in single-stage revision ACL-R in case of recurrent instability after DIS depends on monobloc position during ACL repair. Primary stability is comparable to aperture fixation in primary ACL-R, if a bone stock of 20 mm is left between the monobloc and the tibial joint line during the initial procedure.

## Introduction

Dynamic intraligamentary stabilization (DIS) of the anterior cruciate ligament (ACL) is an innovative surgical technique that aims to repair the ACL in case of an acute tear [1, 2]. Favorable clinical and functional scores were published up to five years after DIS by different authors in the past years [3–8]. Nevertheless, the rate of recurrent instability –due to insufficient healing of the ACL or a re-injury has been described to be amongst 8 and 17% [3, 5–10].

The surgical treatment of recurrent ACL instability is a challenging procedure: the history of previous ACL reconstruction (ACL-R) including a previously harvested tendon as well as the position of an eventual widened bony tunnel can compromise the graft fixation [11–13]. In both primary and revision ACL-R, the majority of orthopedic surgeons’ rely on a hybrid fixation of the ACL graft at the tibial side. Therefore, most commonly an aperture fixation with an interference screw (IFS) and an additional extracortical fixation is used [14]. The diameter of the tibial tunnel and the IFS are chosen depending on the diameter of the tendon graft, which is usually between 6 and 9 mm. In revision surgery of the ACL, a two-stage procedure is often necessary in order to restore the bony anatomy prior to performing a revision reconstruction of the ACL with anatomic bone tunnel placement [11, 13].

**Fig. 1:**
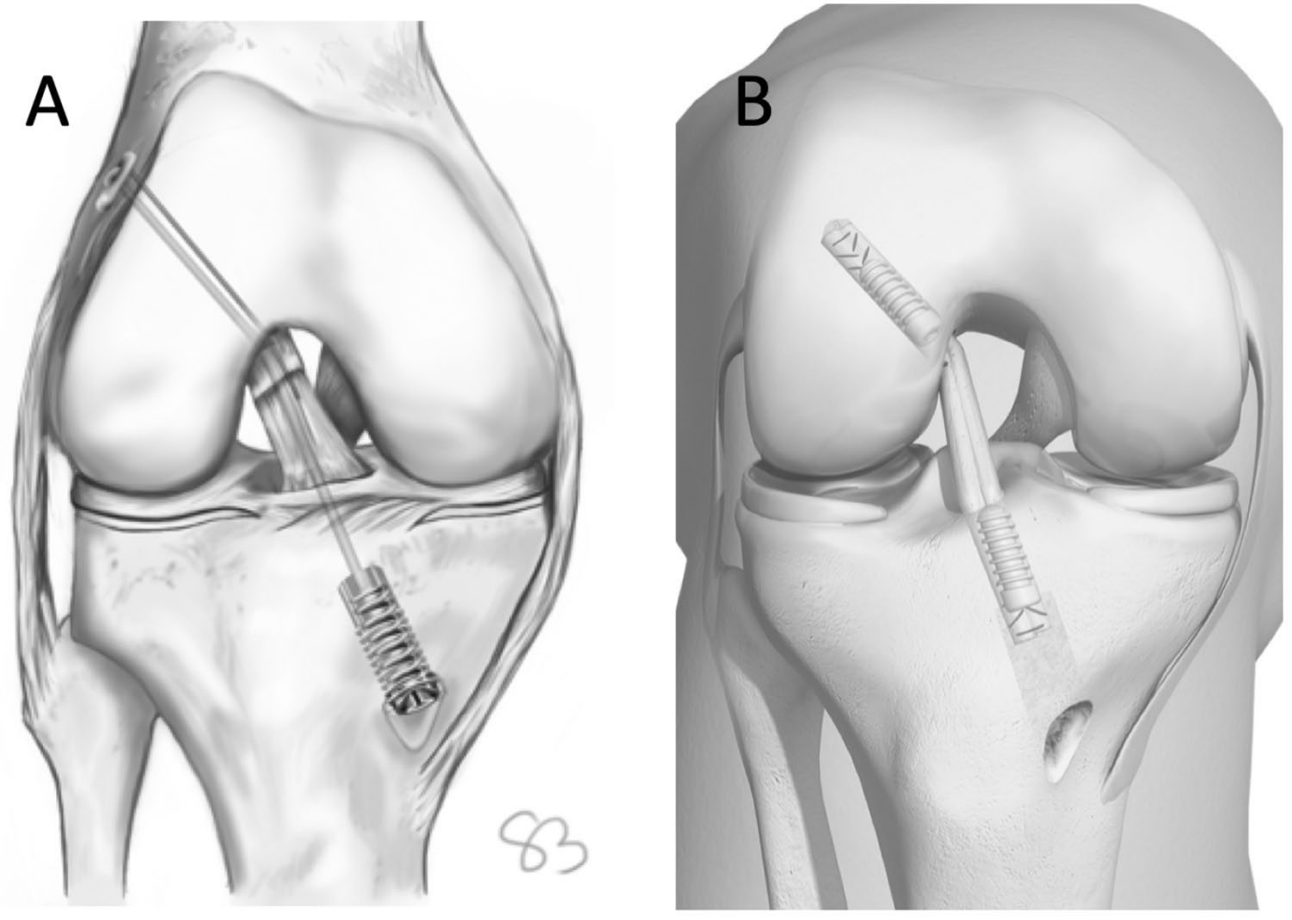
The distance between the cylindrical monobloc (10 × 30 mm) and the tibial joint line in ACL repair with DIS (**A**) should be approximately 20 mm; in the revision situation, an anatomical tibial tunnel for graft fixation could be drilled through the bone void that is left after monobloc removal (**B**)

In case of recurrent instability after DIS the configuration of the pre-existing bone tunnels differs to those of a previous ACL-R: at the lateral femoral condyle the bony tunnel is situated in the anatomical footprint of the ACL with an diameter of 2.3 mm [2]. At the proximal tibia, the intraarticular portion of the bony tunnel is 2.3 mm wide and is located slightly posterior to the ACL insertion. At the distal portion, insertion of the monobloc of the DIS technique requires a drill hole of 10 × 30 mm [2]. In case of a removal of the monobloc and the polyethylene suture a bipartite hole with a diameter of 2.3 mm proximally and 10 mm distally at the proximal tibia is left (Fig. 1) [29]. This configuration of the tibial tunnel might compromise the stability of the aperture fixation in single-stage revision surgery.

The purpose of this study was to evaluate the primary stability of tibial IFS fixation in single-stage revision ACL-R after DIS dependent on the distance between the proximal end of the monobloc and the articular surface during initial ACL repair. It was hypothesized that primary stability of single stage revision would be equal to native ACL-R if a distance of 20 mm was left between the implant and the joint line during ACL repair.

## Materials and methods

The present in-vitro study was conducted according to the ARRIVE guidelines for pre-clinical animal studies. Porcine specimens were obtained from a local butcher—ethics approval was not required by the institutional review board of our institute. The porcine knee specimens were gently defrosted, dissected and mounted in a cylindrical container using polymethyl methacrylate bone cement (Technovit, Heraeus Kulzer, Germany), which was firmly fixed to the socket of the material testing machine.

In the control group (ACL-R, *n* = 15) tibial aperture fixation of a tendon graft with an IFS was performed adapted to the technique described by Petersen et al. [15]: a Kirschner wire was located in anatomical position of the tibial ACL footprint and a perforated 8 mm drill bit was used to create a tunnel of the same diameter. The tibial ACL aimer (Karl Storz, Tuttlingen, Germany) was used to assure a length of the drill hole of 50 mm with an angle 60° to the tibial joint line, starting from the anteromedial cortex of the proximal tibia. Fresh frozen porcine flexor tendons of 150 mm in length were sutured at the free ends and pulled into the tibial tunnel as a double loop with a total diameter of 8 mm. Previously marked lines at 20 and 25 mm distance from the loop enabled to create a tendon-to-bone interface of 20 mm of length.

Aperture fixation of the graft was performed using an IFS of 8 × 23 mm (Karl Storz, Tuttlingen, Germany) according to the user manual for the screw. A nitinol wire was inserted first into the tunnel guiding the screw from the anteromedial cortex to a subchondral position at the articular portion of the tunnel. The position of the screw was controlled macroscopically from both ends of the bony tunnel. Extracortical fixation was not performed in order to exclusively evaluate aperture fixation properties.

**Fig. 2:**
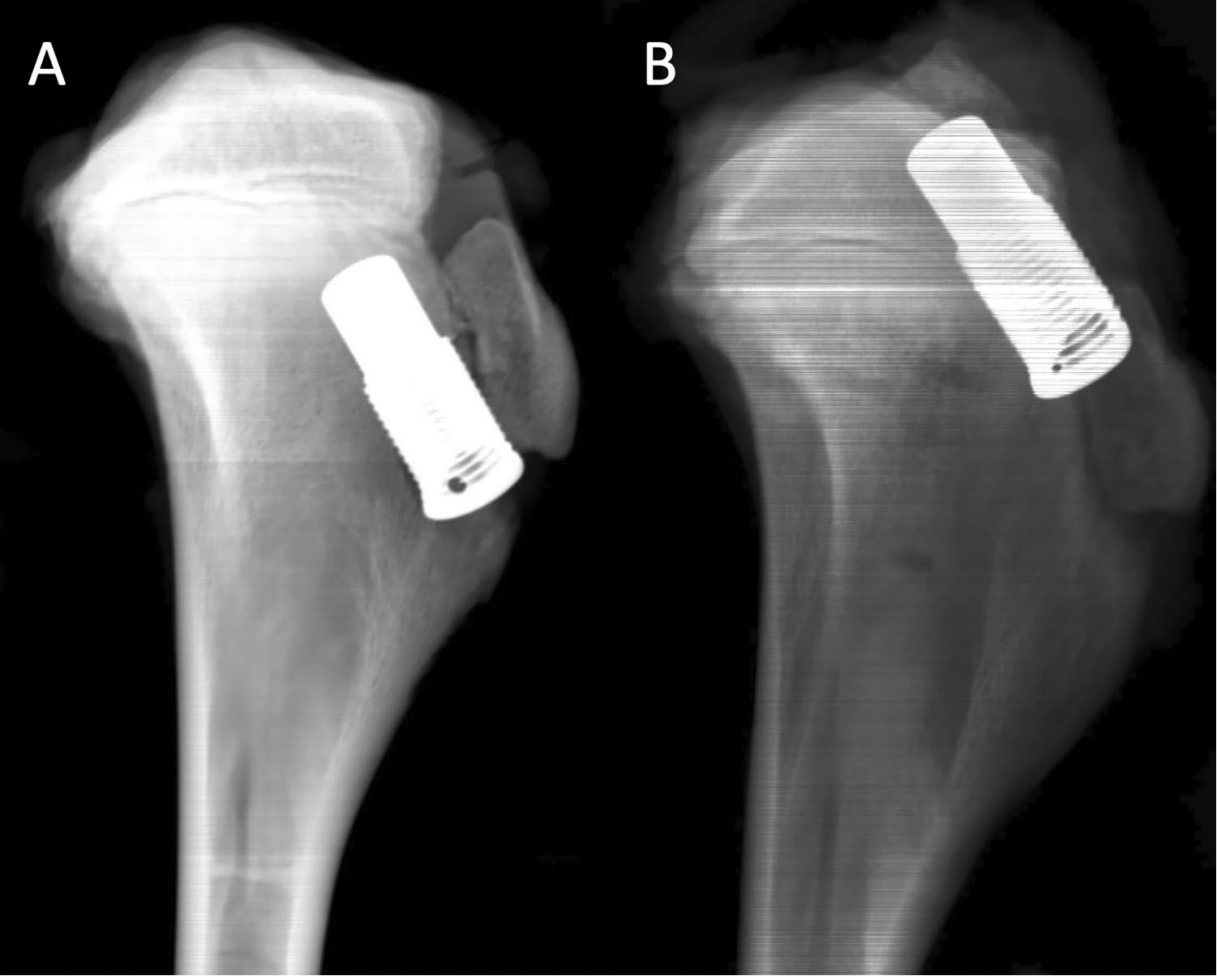
Lateral X-ray control of a porcine tibia after DIS with a distance of 20 mm between the implant and the tibial joint line in group R-DIS1 (**A**) and a distance of 5 mm between the implant and the tibial joint line in group R-DIS2 (**B**)

**Fig. 3:**
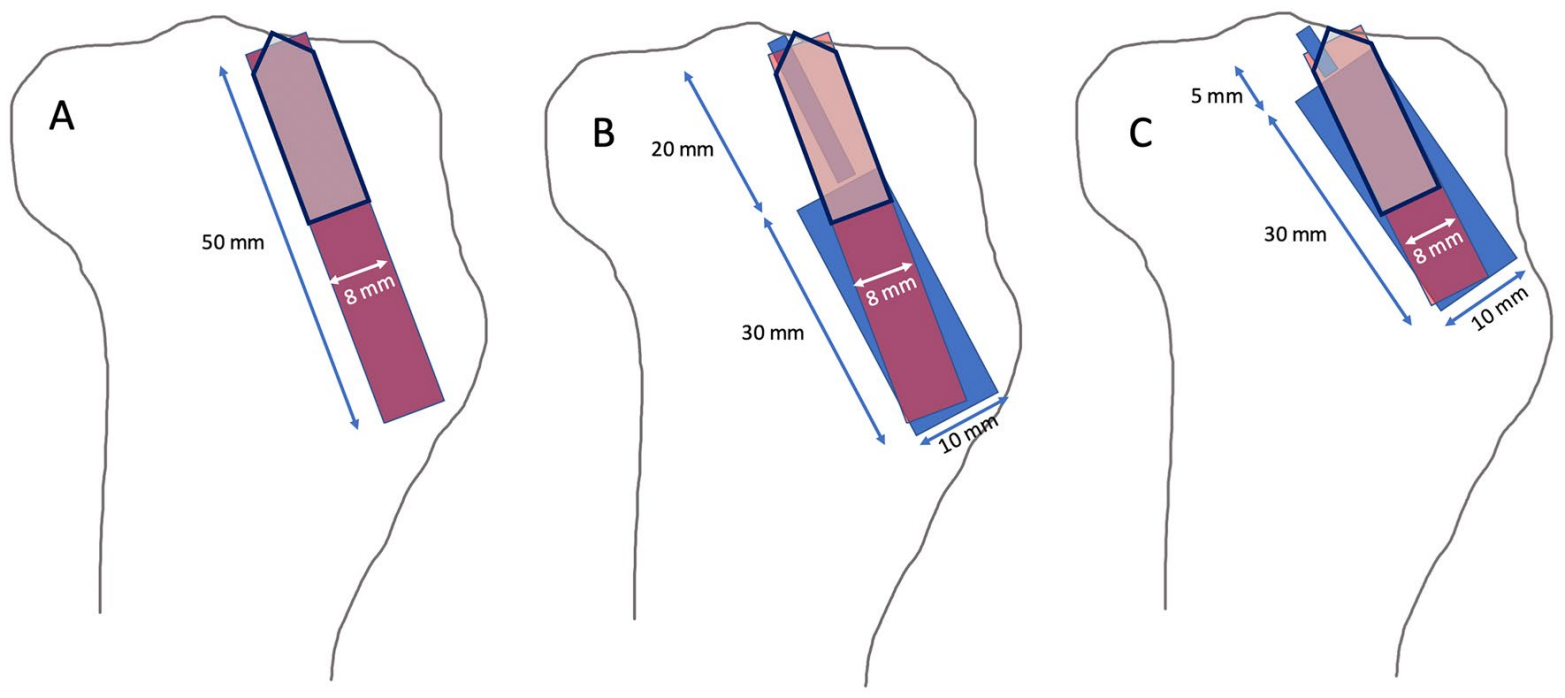
Illustration of tibial tunnel creation with a diameter of 8 mm for ACL-R in each group: native ACL-R (**A**) and single-stage revision in group R-DIS1 (**B**) and R-DIS2 (**C**); aperture fixation of the ACL graft with IFS is represented by black outline, the blue area in B and C is representing the bone defect after removal of the monobloc

In both intervention groups (*n* = 30) DIS was performed using the original implants and instruments of the Ligamys^®^ technique (Mathys medical, Bettlach Switzerland) [2]. Therefor a Kirschner wire was placed from the anteromedial cortex of the tibia with an angle of 60° to the tibial joint line exiting the intraarticular aspect slightly posterior to the anatomic insertion of the ACL. Length of the intraosseous portion of the wire was 50 mm in group R-DIS1 (*n* = 15) and 35 mm in group R-DIS2 (*n* = 15). The distal portion of the tibial tunnel was drilled to a diameter of 10 mm at a length of 30 mm from the cortex and the monobloc was inserted [2]. The wire was removed and the position of the monobloc was controlled in anterior–posterior and lateral x-rays (Fig. 2). The distance between the implant and the tibial joint line was 20 mm in group R-DIS1 and 5 mm in group R-DIS2. The implant was removed using the original instruments for implant removal of the monobloc. Afterwards single-stage revision reconstruction of the ACL was performed in a technique adapted to the procedure described above for the control group. An 8-mm drill hole was created between the distal entry point of the existing two-stage tunnel and the anatomic ACL footprint (Fig. 3). The same diameter of fresh frozen porcine flexor tendons (double loop with a total diameter of 8 mm) and IFS (8 × 23 mm, Karl Storz, Tuttlingen, Germany) as in the control group were used for the single stage revision in both intervention groups. Again, extracortical fixation was not performed.

### Load to failure

A servo-hydraulic Zwick/Roell uniaxial materials testing machine (Z005-TN2A, Zwick/Roell, Ulm, Germany) was used for load-to-failure testing. The free ends of the graft were fixed to the testing machine using a cryoclamp, leaving 30 mm of free graft between the clamp and the bone tunnel and creating an angle of 30° between the bone tunnel and the force vector.

The test protocol included one load of 80 N for preconditioning followed by load-to-failure with a speed of 25 mm/min. Elongation and load were recorded continuously. The mode of failure was macroscopically documented.

### Statistical analysis

A power analysis a priori showed that a sample size of at least 14 specimen per group would lead to a power of 80% to detect a difference of 50 N between means at the f = 0.5 level (half a standard deviation [SD]) based on the standard deviations found in load-to-failure testing of revision ACL reconstruction in a porcine knee model [16].

For statistical analysis, one-way ANOVA was performed in order to detect statistically significant differences between results of each group. Post hoc Bonferroni test was used to determine *p* values between single groups. A *p* value less than 0.05 was required to identify significant differences. The results are presented as mean values and SD. Statistical analysis was performed using IBM SPSS Statistics 26 (IBM, Armonk, NY, USA).

## Results

Load to failure was 454 ± 111 N in the R-DIS1 group and 405 ± 105 N in the primary ACL-R group (*p* = n.s.). In group R-DIS2 load to failure was 154 ± 71 N, which was significantly lower than in R-DIS1 (*p* < 0.001) and ACL-R (*p* < 0.001).

Elongation between the preload and load to failure was comparable between the R-DIS1 group and the ACL-R group (n.s.), whereas a significantly higher elongation was found in group R-DIS2 in comparison to R-DIS1 (p < 0.05) and ACL-R (p < 0.01).

**Fig. 4:**
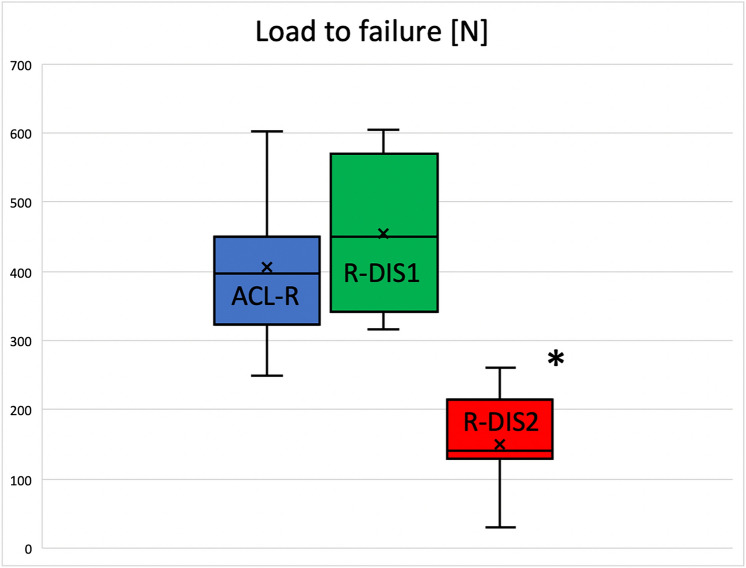
Load to failure in each group presented as boxplots representing range, upper and lower quartile, median (line) and mean (*x*): a significant difference (*) was found between R-DIS2 in comparison to R-DIS1 (*p* < 0.001) and ACL-R, respectively (*p* < 0.001)

Stiffness was determined by the slope of the linear portion of the load displacement curve and was found to be comparable between the R-DIS1 and the primary ACL-R group (*p* = n.s.). Stiffness was significantly lower in group R-DIS2 in comparison to group R-DIS1 (*p* < 0.001) and group ACL-R (*p* < 0.001) (Fig. 4 and Table 1).

**Table 1:** Results as mean ± SD

	ACL-R	R-DIS1	R-DIS2
Load to failure [N]	405 ± 105	454 ± 111	154 ± 71*
Elongation [mm]	7.5 ± 2,3	9.9 ± 3,6	14.9 ± 8.1*
Stiffness [N/mm]	63.4 ± 18,7	73.5 ± 32.8	15.7 ± 4.6*

^*^Is representing a statistically significant difference in comparison to both, ACL-R and R-DIS1, respectively

Mode of failure was graft slippage between the IFS and the tibial tunnel in every specimen. No failure was detected, neither at the tendon graft or the porcine tibia itself, nor at the fixation of the graft or the bone to the testing machine.

## Discussion

The most important finding of this study was that primary stability of tibial aperture fixation in single-stage revision surgery in case of failure of ACL repair with DIS is dependent on the bone stock that is left between the implant and the tibial joint line in the initial surgery. Primary stability in native ACL-R was not superior in comparison to single-stage revision, if a bone stock of 20 mm was left between the monobloc and the tibial joint line in ACL repair with DIS. These findings underline the importance of the monobloc position with respect to the joint line during the initial procedure of ACL repair using DIS in regard to, if needs be, an ACL revision surgery.

Primary stability of tibial of soft tissue graft fixation with an IFS has been assessed in several biomechanical studies; ultimate strength has shown to be between 360 and 1200 N in biomechanical testing, with graft slippage between the IFS and the bony tunnel being the typical mode of failure [16–21]. Maximum load of IFS fixation has proven to be dependent on bone density, screw length and ratio of screw-tunnel-diameter [16–21]. Furthermore, it has been shown that primary stability of tibial IFS fixation is inferior in the revision setting after ACL-R, when a preexisting semi-anatomical tunnel or a tibial tunnel widening is present [16]. As shown in Fig. 3 the bony configuration in group R-DIS2 of the present study corresponds to the revision setting in which the presence of a semi-anatomical tunnel is leading to a bony defect with a higher diameter than the newly created tunnel in case of recurrent instability after ACL-R. Accordingly, primary stability in this group was significantly inferior to native ACL-R (*p* < 0.001). The bony defect left after removal of the monobloc in group R-DIS1 was located 20 mm distally to the tibial joint line and therefore compromised the creation of an anatomical tibial tunnel in single-stage revision in the distal portion only. Primary stability of aperture fixation in this group was equal to primary ACL-R (n.s.) and correspondingly did not differ from primary stability of native ACL-R found in the literature [16, 19, 21, 22].

Soft tissue and bone-patellar tendon-bone (BTB) autografts are equally used in revision ACL-R, with allografts being a common graft option in ACL-R surgery especially in Anglo-American cohorts [11, 23]. Although the bone to bone healing of BTB autografts is considered advantageous in the case of larger bone defects, there is a lack of data comparing the outcome of revision ACL-R in regard to graft choice [23–25]. However, inferior results and knee function have been reported after revision ACL-R in comparison to primary ACL-R independent on graft choice [26], whereas there are no clinical data regarding the outcome of revision ACL-R in the case of recurrent instability following ACL repair.

In order to achieve sufficient primary stability of a soft tissue graft in revision ACL-R, the configuration of the bony tunnels is an important factor to consider when opting for a single- or two-stage revision surgery [27, 28]. In the DIS technique, the distal portion of the tibial tunnel is drilled to a diameter of 10 mm at a length of 30 mm [2]. In contrast to revision surgery after ACL-R, no concern about tunnel widening has been reported so far after DIS [29, 30]. Therefore, when performing ACL repair using the DIS technique the distance between the cylindrical implant site and the tibial joint line is decisive in the revision surgery setting: If a distance of 20 mm or more is left between the monobloc and the tibial joint line, an anatomic bone tunnel can be created in revision ACL-R without concerns regarding confluent tibial tunnels or a lack of bone stock for graft fixation in the subchondral portion of the tunnel. If the distance between the DIS implant and the joint line is lower than 20 mm, a two-stage revision surgery with bone grafting of the defect should be considered, corresponding to the established procedure in revision ACL-R in case of recurrent instability after previous ACL-R [13].

Results of this study are of clinical relevance, considering that ACL repair is performed with increasing frequency in cases of acute ACL injury [31]. While functional and subjective outcomes have shown to be equal to ACL-R there seems to be a slightly higher rate of recurrent instability after ACL repair (8–17%) in comparison to ACL-R [3, 5–10]. Therefore, the ability to opt for a single-stage revision surgery in recurrent ACL instability is an important consideration.

The present study is the first to assess primary stability of tibial aperture fixation after removal of the monobloc of DIS and its results support the option of a single-stage revision including removal of the implant and ACL-R without a bone graft, if there is a bone stock of 20 mm proximal to the implant. It should be taken into account, that additional extracortical fixation further increases primary stability in both primary and revision reconstruction [21].

The results of this study cannot be transferred to the clinical setting without a careful interpretation of this study’s limitations. Porcine tibiae and porcine flexor tendons were used to simulate the tibial fixation of ACL grafts. Specimen were liberated from surrounding soft tissue in order to achieve accuracy in creating the tibial tunnel and therefore increasing internal at the cost of external validity. The biomechanical testing was performed in a porcine knee model as a simulation of the forces acting at time point zero, biologic factors and graft healing were not taken into account. Nevertheless, it has been shown that porcine tendons have similar characteristics to the human semitendinosus tendon [32]. They are therefore used as a xenograft in ACL-R by some authors [33]. Further, it has been shown that the porcine knee best mimics the anatomy and biomechanics of the human knee in comparison to other animal models [34].

## Conclusion

Primary stability of tibial aperture fixation in single-stage revision ACL-R in case of recurrent instability after DIS depends on monobloc position during ACL repair. Primary stability is comparable to aperture fixation in primary ACL-R, if a bone stock of 20 mm is left between the monobloc and the tibial joint line during the initial procedure. Therefore, single-stage revision is a considerable option in these cases. In the future, in vivo studies a required to confirm the results of this study.
